# Management of medullary thyroid cancer based on variation of carcinoembryonic antigen and calcitonin

**DOI:** 10.3389/fendo.2024.1418657

**Published:** 2024-10-10

**Authors:** Bo Wang, Jie Huang, Li Chen

**Affiliations:** ^1^ Department of Paediatric Surgery, Tianjin Medical University General Hospital, Tianjin, China; ^2^ Department of Respiratory and Critical Care Medicine, Tianjin Medical University General Hospital, Tianjin, China; ^3^ Medizinische Klinik und Poliklinik IV, Klinikum der Universität München, Ludwig-Maximilian University of Munich, Munich, Germany

**Keywords:** medullary thyroid carcinoma (MTC), carcinoembryonic antigen (CEA), calcitonin (Ctn), perioperative management, follow-up

## Abstract

Carcinoembryonic antigen (CEA) and calcitonin (Ctn) are pivotal biomarkers in the diagnosis and management of medullary thyroid carcinoma (MTC). However, their diagnostic reliability in perioperative period remains a topic of ongoing debate. This review synthesizes researches on perioperative fluctuations in CEA and Ctn levels, and evaluates the impact of their different combinations on MTC diagnosis, treatment decisions, and prognosis. Our findings highlight it is crucial to understand and interpret the various combinations of CEA and Ctn fluctuations within a clinical context. Furthermore, to reduce diagnostic errors and improve patient outcomes, we recommend follow-up diagnostic and treatment protocols designed to address the potential pitfalls associated with the use of these biomarkers.

## Introduction

Medullary thyroid carcinoma (MTC) is a neuroendocrine tumor originating from the parafollicular cells of the thyroid gland and accounts for 13.4% of thyroid cancer-related mortality (1, 2). Although carcinoembryonic antigen (CEA) and calcitonin (Ctn) are considered crucial biomarkers for MTC, emerging evidence has raised concerns about their diagnostic reliability, especially given that both markers can be elevated in both benign and malignant conditions. For instance, slight increases in Ctn may be seen in patients with C-cell hyperplasia, autoimmune thyroiditis, chronic kidney disease, hyperparathyroidism, and certain lung and neuroendocrine tumors, potentially resulting in false-positive or false-negative outcomes (3)​. Similarly, CEA levels can be influenced by malignancies such as colorectal cancer, breast cancer, and non-small-cell lung cancer (NSCLC), as well as benign factors like liver and biliary dysfunction (4, 5). Additionally, smoking can further elevate CEA levels, complicating the accuracy of diagnosis in clinical practice (6)​.

In response to these challenges, research has explored alternative biomarkers such as procalcitonin, chromogranin A (CgA), and synaptophysin (Syn) (7). However, considering the cost-effectiveness required in public healthcare systems, CEA and Ctn remain the most widely used biomarkers during the perioperative period. While CEA alone lacks specificity for MTC, its combined use with Ctn significantly enhances diagnostic sensitivity (8–10).

Given the limitations of these two biomarkers, it is crucial to understand and interpret the various combinations of CEA and Ctn fluctuations within a clinical context. This review consolidates recent findings on perioperative changes in CEA and Ctn levels and evaluates the impact of their different combinations on MTC diagnosis, treatment decisions, and prognosis.

## Preoperative CEA and Ctn variations

In MTC patients, preoperative serum levels of CEA and Ctn display various patterns of alteration, which can be broadly classified into four categories: 1) Concurrent elevation of both CEA and Ctn; 2) Elevated Ctn with normal CEA; 3) Elevated CEA with normal Ctn; and 4) Normal levels of both biomarkers. **Table 1** summarizes case reports illustrating these patterns. It is important to clarify that in this context, the terms “preoperative” and “postoperative” specifically refer to the timing of surgery for MTC, rather than for other malignancies.

**Table 1:** Clinical characteristics of preoperative MTC cases described in the literature.

Type	Author	Year	Patients Enrolled	Gender	Age	Ctn/(upper limit)	CEA/(upper limit)
1	Vasiliki Daraki (11)	2012	1	Female	45	603 pg/mL (< 11.5 pg/mL)	40.73 ng/mL (< 5 ng/mL)
	Tracy S Wang (12)	2008	1	Male	68	38 pg/mL (< 10 pg/mL)	56.7 ng/mL (< 2.5 ng/mL)
	K A Brzezinska (13)	2023	1	Female	51	8643 pg/mL (< 5 pg/mL)	86.2 ng/mL (< 3 ng/mL)
	Mohamed Hassan (14)	2021	1	Male	38	>585.200 pmol/L	503.0 μg/L
	P D Papapetrou (15)	2012	4				
	Sami Akbulut (16)	2011	1	Male	76	10.5 (< 10 pg/mL)	386 (< 5.2 ng/mL)
	Deepak Abraham (17)	2010	2				
	Xin Nie (18)	2023	1	Female	53	172 pg/mL (< 6.4 pg/mL)	87.5 ng/mL (< 5 ng/mL)
	Regis Cohen (19)	2009	1	Male	73	454 pg/mL (< 10 pg/mL)	9.4 ng/mL (< 5 ng/mL)
	Sema Sezgin Göksu (20)	2014	1	Male	60	3,752 pg/mL (<10 pg/mL)	146 ng/mL (< 5 ng/mL)
	Shih-Wei Chen (21)	2017	1	Male	56	843 ng/L	184 μg/L (< 5 μg/L)
	Alexandre Lugat (22)	2021	1	Male	70	47 ng/L (< 10 ng/L)	47 μg/l (< 5 μg/L)
	Aires Martins (23)	2018	1	Female	73	781.3 (< 6.4 pg/mL)	56.2 ng/mL (< 5 ng/mL)
	Xiao-xiong Gan (24)	2024	1	Male	56	>2000 pg/mL	199 ng/mL
2	Han Zhang (25)	2022	77				
	Amitabha Saha (26)	2022	1	Female	43	406 (< 10 pg/mL)	Normal
3	Gang Zhang (27)	2019	1	Female	48	7.12 pg/mL (< 18 pg/mL)	5.8 pg/mL (< 5 pg/mL)
	L Giovanella (28)	2008	1	Female	43	4.7 pg/mL (< 10 pg/mL)	12.8 ng/mL (< 5 ng/mL)
	Janta Ukrani (29)	2023	1	Female	81	< 2 ng/mL	17 ng/mL
4	José Miguel Dora (30)	2008	1	Male	43	4 ng/L (< 12 ng/L)	0.78 μg/L (< 3.4 μg/L)
	A H Redding (31)	2000	1	Female	31	8.2 pmol/l (7.3-43.9 pmol/l)	<0.5 ng/mL (< 5 ng/mL)
	Wania Rafaey (32)	2022	1	Female	55	<2 pg/mL (< 5 pg/mL)	Normal
	Daisy V Alapat (33)	2011	1	Female	16	4.0 pg/mL (< 4.6 pg/mL)	<1 ng/mL (< 5 ng/mL)
	Maria Teresa Samà (34)	2016	10				
	Lorena Licata (35)	2022	1	Male	39	4 pg/L	<3.7 ng/mL (< 5 ng/mL)
	Patrícia F Baptista (36)	2022	1	Male	64	< 12.5 pg/mL (< 20 pg/mL)	4.1 ng/L (< 5 ng/L)
	Karin Frank-Raue (37)	2013	7				
	M Bockhorn (38)	2004	1	Female	50	0.8 pg/mL (< 4.6 pg/mL)	Normal
	V Chernyavsky (39)	2011	1	Female	40	2.1 pg/mL (< 5 pg/mL)	< 0.5 ng/mL (< 2.5 ng/mL)
	Erika F Brutsaert (40)	2014	1	Female	39	< 2 pg/mL (< 6 pg/mL)	3.1 ng/mL (< 5.2 ng/mL)
	Atsuko Kasajima (41)	2016	1	Female	48	29 pg/mL	1.3 ng/mL
	K W Schmid (42)	1998	4				
	C Gambardella (43)	2019	4				
	Z Jingzhu (44)	2022	1	Female	65	< 2 pg/mL(< 8.4pg/mL)	1.62 ng/mL

Type: 1) Concurrent elevation of both CEA and Ctn; 2) Elevated Ctn with normal CEA; 3) Elevated CEA with normal Ctn; and 4) Normal levels of both biomarkers. Note: The absence of an upper limit or the use of “normal” indicates that a specific value is not provided in the original text.

Firstly, the most common pattern observed is the concurrent elevation of both Ctn and CEA, as reported in previous studies (11–18). R. Cohen (19) et al. reported a case of a 73-year-old male who developed a new obstruction and elevated CEA levels after colon cancer surgery. A PET-CT scan showed increased FDG uptake in the right thyroid lobe, and further tests confirmed elevated serum Ctn levels. A subsequent total thyroidectomy revealed a 14 mm MTC in the right thyroid lobe. Similar cases of post-colorectal cancer surgery elevations in CEA, later diagnosed as MTC, have been documented by other authors (20–23). Additionally, comparable findings have been reported following gastric cancer surgery. Gan et al (24). described a case involving a 56-year-old male who presented with elevated serum CEA levels after gastric cancer surgery. After ruling out gastric cancer recurrence or metastasis, his serum Ctn levels were found to exceed 2000 pg/mL, ultimately leading to the diagnosis of MTC.

Secondly, the occurrence of elevated Ctn with normal CEA values is frequently observed in clinical practice and represents the second most common pattern of biomarker variation (25, 26).

A third subset of patients presents with elevated preoperative CEA levels despite normal Ctn levels, which may suggest a potential tumor dedifferentiation. In a case reported by Gang Zhang et al. (27), a 48-year-old female with hereditary MTC exhibited elevated CEA levels but normal serum Ctn levels. They identified a somatic biallelic loss of the Dicer 1 gene in this patient, which may explain the unusually normal Ctn. Similarly, Giovanella et al (28). described a 43-year-old female patient with elevated preoperative CEA, yet normal Ctn and CgA levels, her postoperative immunostaining, however, revealed positivity for Ctn, CgA, and CEA. Postoperative elevation of CEA with normal Ctn levels has also been observed in patients with a history of surgeries for non-MTC malignancies, leading to a subsequent diagnosis of MTC. Janta (29) reported an 81-year-old breast cancer patient with persistently elevated CEA levels for 15 years post-surgery, normal Ctn levels, and symptoms including weight loss, fatigue, and joint pain. A PET-CT scan detected a lesion, and subsequent pathology confirmed MTC.

Finally, approximately 12% of MTC cases are classified as non-secretory or dual-negative MTC, characterized by normal serum levels of both Ctn and CEA despite the presence of the tumor (30). In a case reported by Allen et al. in 2000 (31), a 31-year-old woman exhibited dual-negative levels of Ctn and CEA prior to surgery. Despite atypical cells being identified on fine needle aspiration (FNA), the final pathology confirmed the diagnosis of MTC. In addition to serologically dual-negative MTC, there are cases where immunohistochemical staining for both Ctn and CEA is also negative, further complicating the diagnostic process. Wania et al. (32) described a middle-aged woman with a large thyroid nodule extending into the superior mediastinum and encasing the right common carotid artery, leading to neck swelling and compression symptoms. Although typical MTC histopathology was observed in the FNA sample, immunohistochemical staining for both Ctn and CEA was negative, while Syn and CgA were positive. Additionally, her serum Ctn and CEA levels remained within normal limits, leading to the diagnosis of “true dual-negative MTC. Other similar cases of dual-negative MTC have been documented by various medical professionals (33–44).

## Changes in CEA and Ctn after surgery

The ideal postoperative outcome for MTC is marked by the normalization of both CEA and Ctn levels. In contrast to the potential challenges posed by preoperative dual-negativity, postoperative dual-negativity is typically regarded as a favorable indicator, particularly for patients with elevated biomarker levels before surgery. However, rare exceptions do occur (45). Tania reported a case of a 65-year-old female with MTC who developed lymph node metastases after surgery but declined further surgical treatment. Despite this, she maintained normal serum Ctn and CEA levels throughout a nine-year follow-up (46). However, in many cases, Ctn and CEA levels may not fully normalize, or may even rise. Generally, three distinct patterns of post-treatment biomarker alterations are observed in MTC patients, reflecting the diversity of clinical outcomes. Case reports illustrating these patterns can be found in **Table 2**.

**Table 2:** Clinical characteristics of postoperative MTC cases described in the literature.

Type	Author	Year	Patients Enrolled	Gender	Age	Ctn/(upper limit)	CEA/(upper limit)
1	Juana Maria Cano (47)	2017	1	Male	64	5967 pg/mL	Increased
	Ibrahim Yildiz (48)	2011	1	Male	61	194 pg/mL (0-30 pg/mL)	133 ng/mL (<3 ng/mL)
	Mitra Niafar (49)	2011	1	Male	40	>2000 pg/mL	98ng/mL (<3 ng/mL)
	Kübra Şahin (50)	2024	1	Male	58	15622 pg/mL	727 ng/mL
3	Li Chen (51)	2020	1	Female	62	4.25 (<5 pg/mL)	9.96 (<5 ng/mL)
	Stéphane Bardet (52)	2022	2				
4	C Gambardella (45)	2019	1	Male	59	< 2.00 pg/mL (< 18.2 pg/mL)	1.49 ng/mL (< 10 ng/mL)
	Tania Tofail (46)	2018	1	Female	65	2.06 pg/mL (<5 pg/mL)	Normal

Type: 1) Concurrent elevation of both CEA and Ctn; 2) Elevated Ctn with normal CEA; 3) Elevated CEA with normal Ctn; and 4) Normal levels of both biomarkers.

The first pattern is characterized by a simultaneous increase in both CEA and Ctn, which is the most frequently observed scenario in clinical practice. This elevation typically suggests persistent or metastatic disease, indicating that the surgery may not have completely eradicated the tumor, or that undetected metastatic sites are actively producing these markers. Juana et al. (47) reported a case of an MTC patient who, despite extensive treatment, had significantly elevated CEA and calcitonin levels. Imaging revealed metastases to the adrenal gland, duodenal bulb, and pancreas. The patient’s condition improved, and tumor markers gradually normalized with somatostatin analog therapy. Ibrahim Yildiz and colleagues (48) described a 63-year-old male MTC patient who, four months post-surgery and radiotherapy, showed a significant increase in tumor markers (Ctn: 194 pg/mL, CEA: 133 ng/mL). Imaging revealed widespread metastases, and the patient succumbed to the disease shortly thereafter. Mitra et al. (49) detailed the treatment of a 40-year-old woman who, despite total thyroidectomy and limited cervical dissection, continued to show persistently elevated CEA and Ctn levels. Further lymph node dissection confirmed metastatic disease, but her tumor markers remained elevated. A subsequent somatostatin receptor scan revealed additional metastatic lymph nodes in the anterior superior mediastinum, prompting further surgical intervention. Similar cases have been continuously documented in the literature (50).

The second pattern is characterized by an increase in Ctn with normal CEA levels. This may occur when the tumor burden is predominantly associated with Ctn-producing cells, without significant activity from cells typically linked to CEA production. This pattern may indicate a partial treatment response, with residual tumor cells remaining active or progressing slowly, it generally suggests a more favorable prognosis compared to the other patterns.

Elevated CEA levels with normal Ctn have also been documented in several publications. Although less common, this scenario may occur when tumor components produce CEA but not Ctn, or when non-thyroidal sources of CEA are involved. In our previous study (51), we reported the case of an elderly female with MTC who exhibited rising CEA levels after total thyroidectomy and central lymph node dissection. Although asymptomatic during follow-up, her CEA levels continued to increase until metastases were identified in the lymph nodes and bones. Moreover, in patients with MTC undergoing targeted therapy, persistent elevation in serum CEA levels alongside declining Ctn levels may occur. Stéphane et al. (52) described two patients who exhibited elevated CEA levels despite normal Ctn after MTC surgery, while responding positively to selpercatinib treatment. However, it is important to recognize that transient fluctuations in tumor markers are common in MTC patients receiving RET inhibitors, these short-term oscillations may not necessarily reflect long-term responsiveness (53).

## Preoperative evaluation based on CEA and Ctn

As previously mentioned, despite the existence of more robust independent prognostic factors and advanced grading systems, such as the International MTC Grading System (IMTCGS) based on mitotic count, Ki67 proliferative index, and tumor necrosis, the most widely used biomarkers remain Ctn and CEA (54–57). But there is ongoing debate about whether routine preoperative screening for Ctn and CEA should be implemented. For instance, in the UK (58), routine Ctn measurement for evaluating thyroid nodules is not recommended due to concerns about high costs and the potential for unnecessary thyroidectomies in patients with benign conditions. In contrast, the American Thyroid Association (ATA) leaves the decision up to the treating physician’s discretion (3). Meanwhile, the German thyroid cancer guidelines advocate for preoperative Ctn screening in all patients undergoing thyroid surgery to ensure early detection and timely treatment of MTC (59). Supporting this approach, conclusions drawn from multi-center retrospective cohort studies conducted in Israel (60) and China (61) also endorse the use of preoperative Ctn screening.

Given the ongoing debate surrounding preoperative screening, the accuracy of Ctn as a diagnostic tool is critically important. This accuracy is closely tied to established threshold levels and the progressive increase in Ctn concentrations. Theresia Weber (62) identified optimal Ctn thresholds for MTC diagnosis, setting the cutoff at 7.9 pg/mL for females and15 pg/mL for male, respectively. In addition, Friedhelm Raue’s research further showed that the positive predictive value (PPV) for diagnosing MTC increases with higher serum Ctn levels: 8.3% for levels between 20-50 ng/mL, 25.0% for levels between 50-100 ng/mL, and 100% when levels exceed 100 ng/mL (63). In cases where Ctn levels are marginally elevated or near critical thresholds, particularly in Ctn-negative MTC due to defective secretion mechanisms, diagnosis can be particularly challenging. Traditionally, diagnosis relied on calcium or pentagastrin stimulation tests, but these have fallen out of favor, pentagastrin is no longer available, and calcium tests are largely abandoned due to high false positive rates (64) and significant side effects (65). A promising alternative is the detection of Ctn Gene-Related Peptide (CGRP), which shows potential as an early diagnostic biomarker for MTC (66, 67).

Furthermore, preoperative serum Ctn levels are closely linked to several critical clinical parameters, including tumor size, lymph node involvement, distant metastasis, as well as sex (68). Saltiki et al. (69) demonstrated a positive correlation between serum Ctn levels and MTC tumor size, with higher Ctn levels generally indicating larger tumors—a finding that has been corroborated by additional studies (70–72). The relationship between Ctn levels and lateral neck lymph node metastasis has been extensively investigated, although the specific cut-off values remain a subject of ongoing debate. Andreas reported that nodal metastasis tends to emerge when basal Ctn levels increase to 10–40 pg/mL (73). Similarly, Wells. et al. (3, 71) emphasized that baseline serum Ctn levels exceed 20 ng/L are generally associated with risk of lymph node metastasis. In contrast, Hyunju’s research found that Ctn levels above 20, 50, and 200 ng/L correspond to the risks of lymph node involvement of 11%, 17%, and 45%, respectively, affecting areas such as the ipsilateral VI and lateral neck, contralateral VI region, and contralateral neck (74). Additionally, other similar studies have also explored different cut-off values (75, 76).

Hyunju’s research also identified a preoperative Ctn threshold of 500 pg/mL as being associated with the presence of distant metastasis in MTC patients (74). Similarly, Han Zhang (25) found that the optimal Ctn threshold for predicting TNM IV was 167.00 pg/mL. It is also crucial to reiterate that a negative preoperative Ctn result does not definitively exclude the possibility of an MTC diagnosis, as previously discussed. Another often overlooked factor is the sex. As highlighted in the study by Theresia et al. (62), and supported by similar research (70, 77, 78), a consistent finding across these studies is the significant difference in Ctn levels between males and females, despite variability in threshold values (70, 77, 78).

Given the close association between preoperative serum Ctn levels and key clinical factors such as tumor size, lymph node involvement, and distant metastasis, Ctn levels play a role in determining the optimal extent of surgical intervention, though this remains a matter of ongoing debate. According to the recommendations of the American Thyroid Association (ATA) guidelines, in cases where no evidence of neck metastases is observed on ultrasound and no distant metastases are present, lateral neck dissection (levels II-V) may be considered based on serum Ctn levels. When preoperative imaging identifies metastasis in the ipsilateral lateral neck compartment but not in the contralateral neck, contralateral neck dissection is recommended if basal serum Ctn levels exceed 200 pg/mL (3, 71). Research by Juez et al (79). suggested that for tumors with RET mutations, prophylactic bilateral neck dissection should be performed when Ctn levels exceed 200 pg/mL. For sporadic MTC, prophylactic bilateral neck dissection is recommended when baseline Ctn levels surpass 600 pg/mL. However, differing opinions exist. The British Thyroid Association guidelines highlight that the impact of Ctn levels on overall survival remains uncertain (80). Additionally, Else et al. found that baseline Ctn levels alone do not reliably predict the necessity for prophylactic lateral neck dissection in MTC patients, underscoring the need for further research to clarify this issue (81).

Although CEA is not a specific biomarker for MTC, it holds value in preoperative evaluation. CEA concentrations of 30 μg/L suggest involvement of central and lateral lymph nodes, while levels above 100 μg/L are strongly associated with extensive lymph node involvement and distant metastases, offering guidance for determining the extent of surgery (82–84). In a retrospective study conducted by Turkdogan et al. (85) involving 33 MTC patients, preoperative CEA levels exceeding 271 ng/mL were linked to more aggressive tumor characteristics and a poorer prognosis, with levels above 500 ng/mL significantly associated with increased mortality rates. Determining the source of CEA secretion (thyroid, nonthyroid, or both) is crucial in diagnosing patients with elevated preoperative CEA.

In preoperative diagnostics, a particular combination warrants attention: CEA and Ctn dual-negative MTC. This subtype may have a low secretory capacity or slower tumor growth, resulting in normal or undetectable levels of CEA and Ctn. Consequently, diagnosis is often delayed until a more advanced clinical stage, leading to a generally poorer prognosis (37). Fuchs et al (57). corroborated this observation, showing significantly shorter survival in Ctn-negative MTC cases compared to Ctn-positive cases (24 vs. 185 months). Similarly, CEA-negative cases demonstrated markedly reduced mean survival compared to CEA-positive cases (52 vs. 186 months). The underlying causes of Ctn negativity remain uncertain but may include impairments in synthesis, storage, or secretion, tumor dedifferentiation, precursor peptides, abnormal Ctn isoforms, variations in synthesis cycles, or differences in detection methods (44). The preoperative diagnosis of non-secretory MTC remains challenging, making a comprehensive evaluation crucial for improving clinical outcomes.

## Postoperative management based on CEA and Ctn

**Figure 1:**
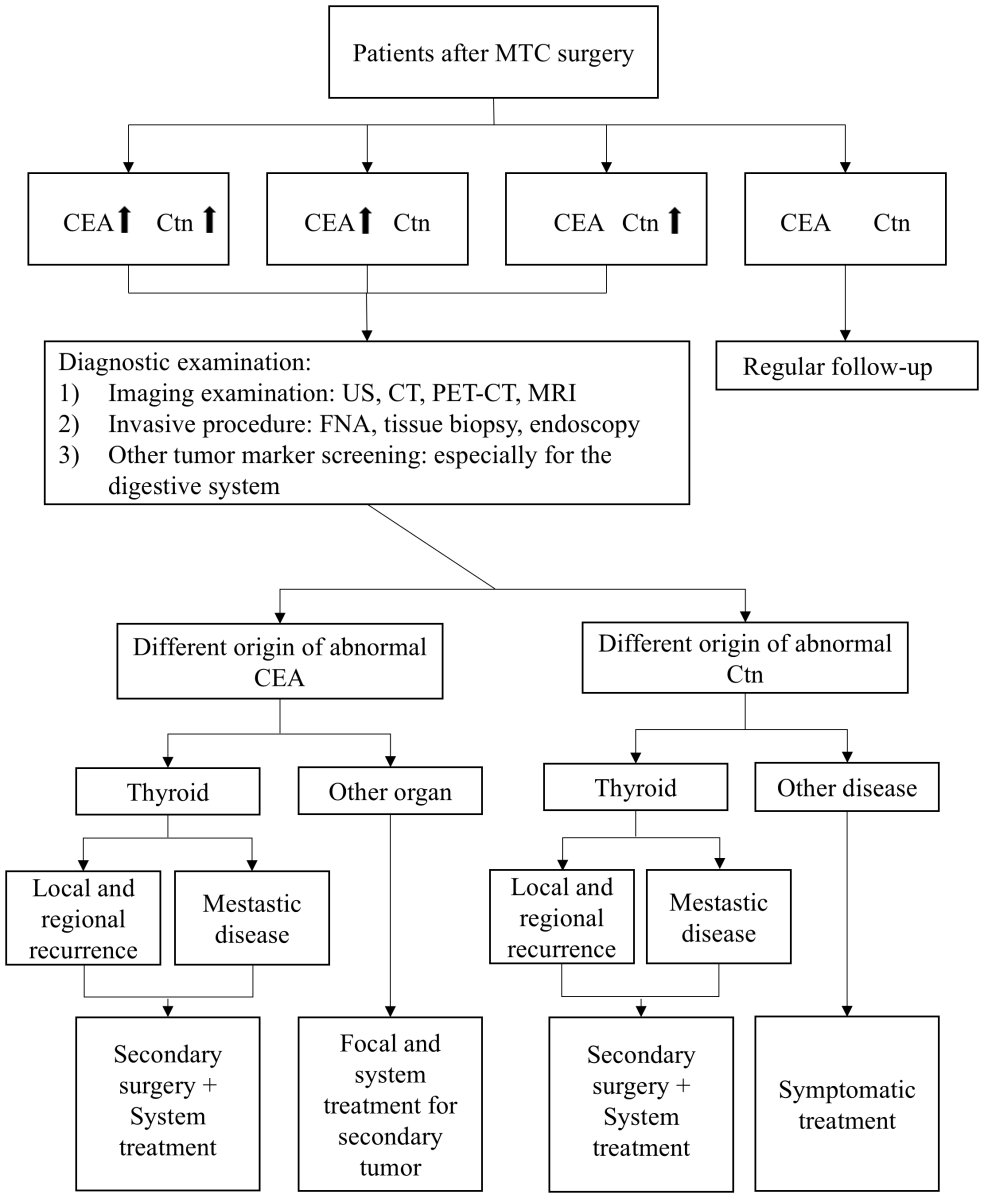
Postoperative follow-up strategies based on changes in CEA and Ctn levels.

The critical role of CEA and Ctn in preoperative diagnosis extends into the postoperative phase, where their continued monitoring is essential for detecting residual cancer and assessing the risk of recurrence. **Figure 1** provides a summary of our recommended postoperative follow-up strategies based on changes in CEA and Ctn levels.

## Prognosis value of CEA and Ctn

In 2008, Michael Tuttle first introduced a dynamic risk stratification system for differentiated thyroid cancer (DTC) (86), which was later adapted in 2013 to address MTC. This modified system incorporates postoperative serum Ctn and CEA levels, alongside imaging studies, to categorize patients into four distinct response categories: excellent, biochemical incomplete, structural incomplete, and indeterminate (87). Research has shown that achieving biochemical remission is associated with a 10-year survival rate of 97.7% (88) and is also the strongest predictor of recurrence-free survival (89). Further studies have refined these findings, showing that within this group, patients who achieved postoperative Ct level normalization (2-10 pg/mL) face a higher risk of disease recurrence compared to those with undetectable Ct levels (below 2 pg/mL) after surgery (90). For patients with a biochemical incomplete response, long-term follow-up is crucial, as 50% of these patients develop structural disease within 10 years (91). Additionally, Cho et al (92). identified a Ctn cutoff value of 29.0 pg/mL as a predictor of structural recurrence, while the ratio of early postoperative to preoperative Ctn levels has also been suggested as a valuable indicator for predicting structural recurrence and biochemical incomplete (93, 94).

Three key time-related metrics—normalization time, testing time, and doubling time—are especially informative in the postoperative management of MTC. Given Ctn’s half-life, Ctn levels generally normalize within one week post-surgery. However, research by Machens et al. (95). suggest that in node-positive MTC patients with preoperative Ctn levels between 500.1 and 1000 pg/mL, the normalization period may extend to two weeks. For patients with preoperative Ctn levels exceeding 1000 pg/mL and with more than ten nodal metastases, normalization may be further delayed. Regarding the timing of initial postoperative Ctn testing. The American Thyroid Association advises testing three months after surgery, while the UK recommends waiting until six months.

Additionally, the Ctn doubling time is a highly prognostic marker. In high-grade MTC (characterized by a mitotic index ≥ 5/2 mm², tumor necrosis, or a Ki-67 proliferation index ≥ 5%) the Ctn doubling time is significantly shorter, averaging 8.51 ± 3.22 months compared to 38.42 ± 11.19 months in lower-grade MTC patients (96). Jacques et al. (97) found that in 65 postoperative MTC patients, a Ctn DT of less than 6 months was linked to 5- and 10-year survival rates of 25% and 8%, respectively. For those with a Ctn DT between 6 months and 2 years, survival rates improved to 92% and 37%. The study by Anne et al. (98) also confirms the role of Ctn doubling time in postoperative evaluation. However, there remains some controversy regarding the specific threshold values. However, it is important to note that even patients with normal or undetectable serum Ctn levels may still carry varying risks of recurrence.

Although CEA is not a specific marker, its decline is faster than that of Ctn, making it a more reliable biochemical indicator for predicting biochemical cure in the postoperative management of MTC (99). Among these indicators, CEA doubling time is valuable for evaluating disease progression and overall prognosis, particularly in patients with advanced-stage disease (15, 97, 98). Moreover, in MTC patients undergoing cytotoxic chemotherapy, an increase in serum CEA levels three months postoperatively serves as a reliable surrogate marker for predicting shorter progression-free survival (PFS) (100).

## Postoperative imaging examination choice

The postoperative detection of residual or recurrent MTC typically relies on conventional imaging techniques such as ultrasound, CT, MRI (101), and 99mTc-DMSA scintigraphy (102).There has been considerable discussion surrounding the choice of appropriate radiotracers to improve diagnostic efficacy based on postoperative changes in CEA and Ctn levels. Among the most frequently studied tracers are 18F-DOPA, 18F-FDG, and 68Ga-DOTANOC. Jiang (103) and colleagues have shown that FDG is particularly effective when Ctn levels exceed 1000 pg/mL (100). However, Pedro’s findings suggest that FDG detects hypermetabolic lesions related to CEA levels but is not correlated with Ctn, whereas 68Ga-DOTANOC is more closely associated with Ctn levels but not with CEA (104). Ana et al (105). reported that DOPA is superior to FDG in detecting and localizing lesions in patients with recurrent MTC, especially in those with negative results on other imaging modalities and with Ctn ≥150 pg/mL or CEA ≥5 ng/mL. Similarly, Marzona’s research supports this, demonstrating that in MTC patients with rapidly increasing Ctn levels during follow-up, DOPA exhibits good sensitivity and complements FDG in detecting metastatic deposits (106). A network meta-analysis (NMA) further corroborates these findings, showing that DOPA clearly outperforms other imaging modalities in detecting recurrent MTC in both patient- and lesion-based analyses, regardless of serum Ctn or CEA levels, or Ctn doubling time (107).

## The treatment principle for recurrence and metastatic lesion

For localized recurrences after surgery, neck exploration combined with selective or comprehensive neck dissection is typically performed to remove the affected lymph nodes. In cases of distant metastases, which commonly include the lungs, liver, bones, and brain, the treatment approach generally involves targeted interventions at the metastatic sites alongside palliative care to manage symptoms. When metastases become too widespread or are not amenable to surgical treatment, the focus shifts primarily to symptomatic palliative care. For instance, liver metastases can be treated with surgical resection, either through open or laparoscopic approaches, or with arterial embolization. The choice of treatment depends on the size and number of metastases, as well as the patient’s liver function and overall health status (108).

## Postoperative molecular targeted therapy

For recurrent MTC, the use of tyrosine kinase inhibitors (TKIs) has emerged as a promising therapeutic strategy. These agents work by blocking the signaling pathways of multiple growth factor receptors, thereby inhibiting tumor cell proliferation and angiogenesis (109). To date, two multi-TKIs have received FDA approval for the treatment of MTC. Vandetanib (110) targets multiple pathways, including RET, VEGFR, and EGFR, and is particularly effective in patients with locally advanced or metastatic MTC. On the other hand, Cabozantinib (111) not only inhibits RET, MET, and VEGFR2 but has also shown efficacy in patients resistant to traditional therapies. Clinical trials, including the ZETA trial for Vandetanib (112) and the EXAM trial for Cabozantinib (113), have demonstrated the ability of both drugs to significantly extend PFS in MTC patients. Additionally, investigational targeted therapies are being developed, including new-generation selective inhibitors of RET mutations, such as selpercatinib (114) and pralsetinib (115). These drugs have shown strong efficacy in patients with RET-mutant MTC.

Furthermore, lenvatinib (116), a multi-target TKI that inhibits VEGFR, FGFR, PDGFR, and RET, has been used as a subsequent line of treatment, particularly in patients who are resistant to Vandetanib or Cabozantinib. Another investigational drug, Surufatinib (117), targets VEGFR, FGFR, and CSF-1R and demonstrates dual action in inhibiting tumor immune evasion and angiogenesis. Though still in clinical trials, Surufatinib has shown promising antitumor activity, especially in Chinese patients with MTC. Similarly, Anlotinib, which targets VEGFR, PDGFR, and FGFR, has proven its efficacy and safety in Chinese MTC patients (118). Despite the significant therapeutic benefits of TKIs in treating MTC, their associated adverse effects cannot be overlooked, like hypertension, gastrointestinal discomfort, skin toxicity, and thyroid dysfunction (119).

## Other therapy options

Alternative therapeutic approaches for advanced MTC include checkpoint inhibitors such as anti-PD-1 and anti-CTLA-4 antibodies, which have shown efficacy in immunotherapy (120). Additionally, recombinant human leukemia inhibitory factor (LIF) targets RET kinase domain mutations, arresting MTC cell growth in the G0/G1 phase, potentially serving as an effective anticancer agent (121). Innovations in cancer vaccines, gene therapy, and pre-targeted radioimmunotherapy are also enhancing the treatment landscape for MTC.

## Conclusions

This review provides a comprehensive synthesis of reported MTC cases involving CEA and Ctn, systematically evaluating the impact of perioperative fluctuations in these biomarkers on diagnosis, treatment decisions, and prognosis. To our knowledge, this is the first systematic study to investigate MTC through the combined analysis of CEA and Ctn changes. Our findings emphasize the critical need to understand and address the variability in these biomarkers, which is essential for developing more precise and individualized treatment strategies that can significantly enhance patient prognosis and quality of life.
